# Myelitis as a side effect of tofersen therapy in *SOD1*-associated ALS

**DOI:** 10.1007/s00415-023-12130-1

**Published:** 2023-12-09

**Authors:** Peter Reilich, Florian Schöberl, Miriam Hiebeler, Matthias Tonon, Albert C. Ludolph, Makbule Senel

**Affiliations:** 1 Present Address: Friedrich Baur Institute at the Department of Neurology, University Hospital, LMU Munich, Ziemssenstr. 1, 80336 Munich, Germany; 2 Present Address: Department of Neurology, University Hospital, LMU Munich, Munich, Germany; 3 Department of Neurology, University Clinic, University of Ulm, Ulm, Germany

Dear Sirs,

Amyotrophic lateral sclerosis (ALS) is a neurodegenerative multisystem disease with predominant involvement of the motor system, motor system with functional impairment [8], and a characteristic pattern of paresis [5].

5–10% of all cases can be attributed to monogenetic causes, most frequently pathogenic variants in the *C9orf72, SOD1, TARDBP,* and *FUS* genes (with decreasing frequency) [2, 9, 13]. *SOD1*-associated forms account for nearly 2% of all ALS cases [9].

Recently, the antisense oligonucleotide tofersen became the first gene-based therapy for ALS to receive accelerated approval in the USA for the treatment of *SOD1*-associated ALS based on the results of the VALOR trial and its open label extension published in 2022 [7]. Tofersen is currently available in many other countries as part of an early access program (EAP).

According to the published data leading to approval [7], tofersen leads to a significant decrease in the concentration of neurofilament light chain (NfL) as a plasma biomarker of neuroaxonal degeneration with good overall tolerability. Serious side effects were reported as aseptic meningitis, lumbar radiculopathy, increased intracranial pressure, papilledema, and myelitis in 7% of all study participants. Among them, myelitis has only been mentioned in one patient [7] who recovered within 3 months with the use of glucocorticoids and plasma exchange, although further details of symptoms and findings are not available.

To the best of our knowledge, here we present the first detailed report on a patient who developed myelitis during therapy with tofersen in the EAP in Germany.

The 56-year-old patient developed generalized fasciculations and atrophic paresis of the left hand over a period of 2 months. Four months after the initial symptoms, the neurological examination revealed paresis of the external rotation of the upper arm, the finger extensors, and the grip strength of the left hand with increased reflexes on all sides. MRI of the brain and the cervical spine did not show any conclusive findings. Electromyography showed active denervation signs in 3 of 4 regions with chronic neurogenic remodeling; nerve conduction velocities were regular. In CSF diagnostics, protein, cell count, IgG, and oligoclonal bands (OCB) were unremarkable. The phosphorylated neurofilament heavy chain (pNfH) in CSF was clearly elevated (2256 pg/ml), as was neurofilament light chain (NfL) in plasma (225 pg/ml). Typically, neurofilament levels in ALS patients are detected in the range of > 560 pg/ml pNfH in CSF and > 45 pg/ml NfL in plasma [10]. Molecular genetic panel diagnostics (*ANG, ANXA11, CHCHD10, FIG4, FUS, SETX, SLC52A2, SLC52A3, SOD1, TARDBP, TBK1, TIA1, TUBA4A, UBQLN2, VAPB; C9orf72, SCA1/2/3, AR*) detected a heterozygous pathogenic class IV variant in the *SOD1* gene (c.341 T > C; p.(Ile114Thr) and a likely pathogenic class 4 variant in the *FIG4* gene (c.122 T > C; p.(Ile41Thr). The family history (parents, 1 younger sister) was negative regarding neuromuscular diseases. Except for psoriasis vulgaris (body surface area < 3%, PASI < 3), there was no past medical history and no regular medication.

**Fig. 1:**
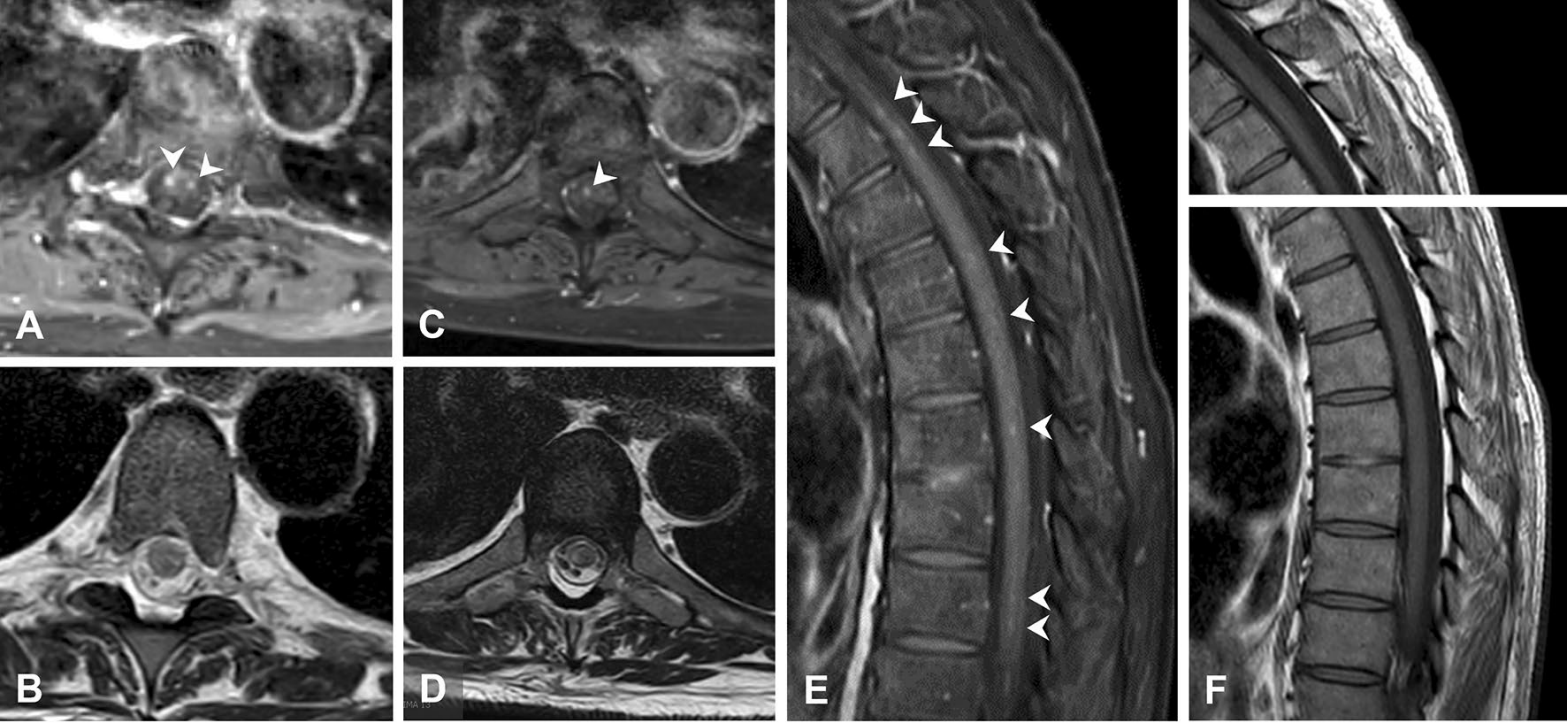
**A**, **C** Examples of low, partly punctate contrast uptake of the spinal cord (white arrows) at two different (T5, T7) thoracic levels in transversal view and **E** in sagittal view at different (T1, T3/4, T5, T7) levels in gadolinium enhanced T1w sequences (see white arrows). The corresponding unenhanced images are, respectively, shown below in T2 TSE (**B**, **D**) and beside in T1w technique (**F**)

Intrathecal therapy with tofersen 100 mg was started 6 months after the onset of initial symptoms. Injections 1–5 (days 1, 14, 28, 55, 84) were tolerated without side effects; the patient noticed a general mild feeling of weakness in the first 2–3 days after injection, which subsided thereafter. Three days after the 6th injection (performed on day 112, CSF conspicuous with 56 cells/µl, elevated total protein 79 mg/dl and positive OCB), generalized myalgias developed, predominantly regarding the lumbar area and lower extremities, and the feeling of restless legs with evening accentuation. Clinical examination (day 116) revealed paresis of foot extensors (4–5/5 MRC) as well as thigh adduction 4–5/5. Plantar response was extensor bilaterally. Oral therapy with prednisolone 20 mg for 2 days provided only a modest benefit with regard to the myalgias, so that from day 3 onwards, it was extended to 40 mg prednisolone and 1000 mg metamizole (3 × daily). A lumbar MRI was able to exclude an intraspinal hematoma and inflammatory contrast uptake of the spinal cord (Fig. 1). On day 122, leg paresis had increased significantly (right foot elevation 3/5, left 4/5, foot depression 4–5/5, hip flexion 3–4/5, hip extension 4–5/5, knee extension 4/5, knee flexion 4–5/5), so that the patient was admitted as an inpatient on day 124.

**Fig. 2:**
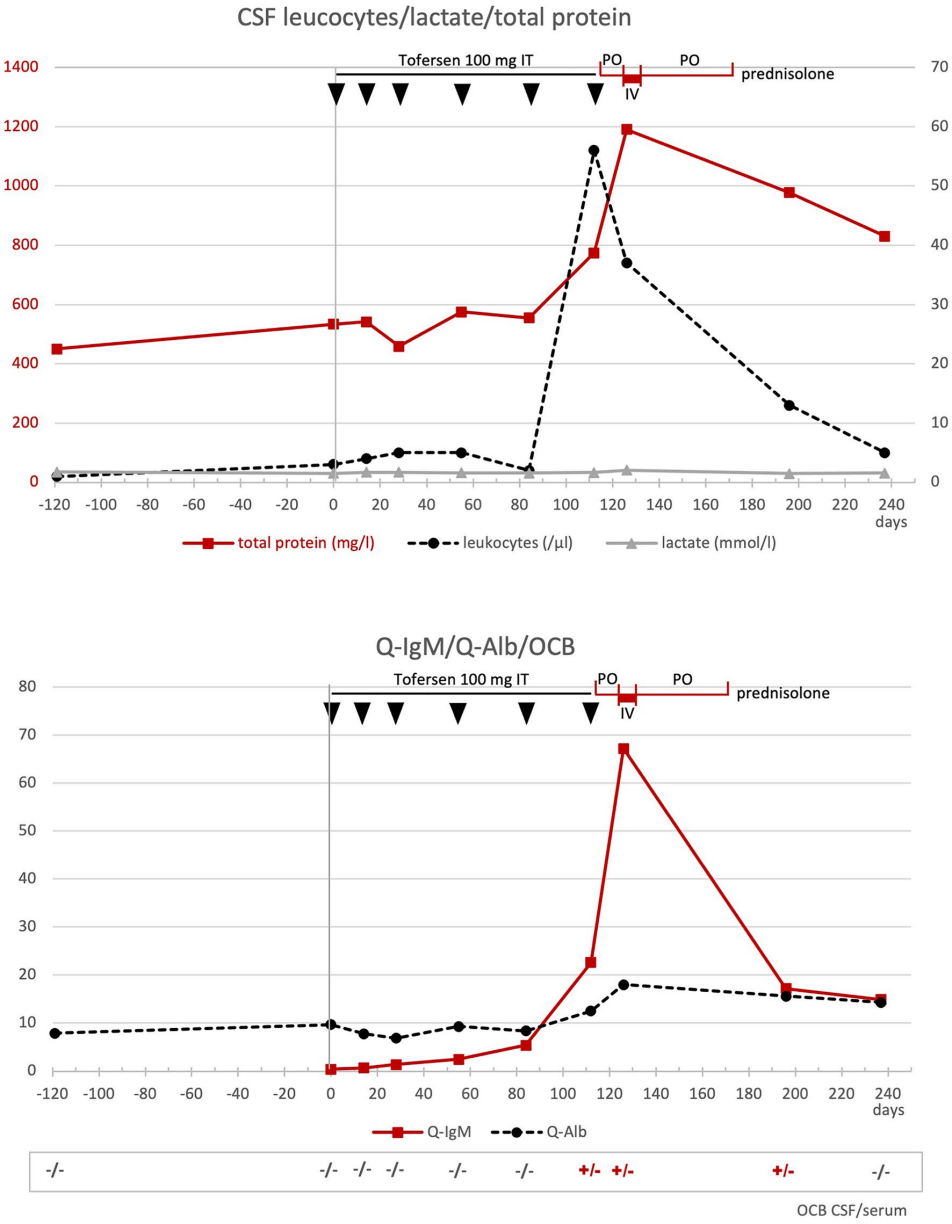
(**top**) CSF values for leukocytes (dashed black line), lactate (gray line), and total protein (continuous red line) are shown over time. [**bottom**]: CSF/serum ratios for IgM (red line) and albumin (dashed black line) over time.Tofersen was administered intrathecally (IT) at days 1 (vertical gray line), 14, 28, 55, 84, and 112 (black arrows). The administration of prednisolone during the period from days 116 to 172 is shown in red on the time line at the top of the graphs (po = oral; iv = intravenous)

**Fig. 3:**
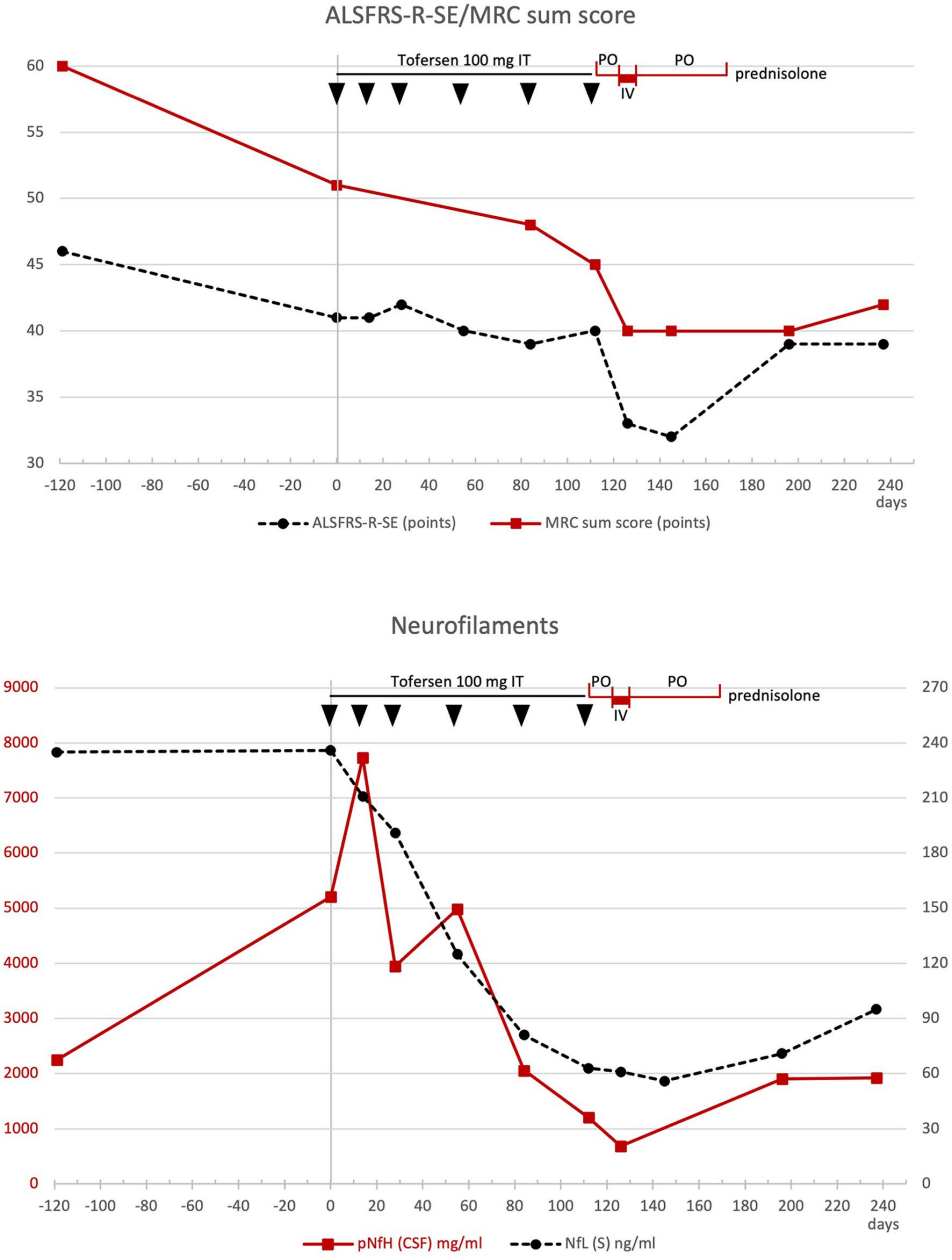
(**top**) Values of the ALSFRS-R-SE (dashed black line) and the MRC sum score (red line) within the clinical course. (**bottom**) Values of the neurofilaments (CSF-pNfH in red, serum NfL in dashed black line) over time. Also shown in the footer are the results of oligoclonal bands (OCB) in CSF/serum with detection of CSF-specific OCB on days 112, 145, and 196. Tofersen was administered intrathecally (IT) at time points days 1 (vertical gray line), 14, 28, 55, 84, and 112 (black arrows). The administration of prednisolone during the period from days 116 to 172 is shown in red on the time line at the top of the graphs (po = oral; iv = intravenous)

The evoked potentials of the tibial nerve showed a symmetrical delay of the P40 responses, the amplitude of the sural nerve was slightly reduced. MRI of the cervical and thoracic spine (day 126) showed low, partly punctiform contrast uptake and edema, consistent with myelitis. Additional CSF diagnostics showed an increase in cell count of 37/µl (lymphocytic), elevated total protein 119 mg/dl, IgM quotient CSF/blood 67.2 and positive CSF-specific OCB (Fig. 2). Multiplex PCR for viral and bacterial agents was unremarkable. Under intravenous therapy with 1.000 mg prednisolone/day for 5 days (days 126–131) followed by an oral reduction regimen for 40 days (days 132–172), starting with 60 mg prednisolone orally, the sensory symptoms and RLS regressed rapidly, while leg weakness regressed much more slowly and showed improvement in MRC sum score [4] only on day 237; whereas, the ALSFRS-R-SE function score [3, 6] indicated functional improvement earlier (day 196) (Fig. 3). On day 237, about 110 days after the start of cortisone therapy, the leg paresis had clearly stabilized, despite the progression of the underlying disease, and the strength had even increased compared to day 122 (foot elevation 4/5, foot depression 4–5/5, hip flexion 4/5, hip extension 4–5/5, knee extension 5/5, knee flexion 4–5/5, on both sides).

There was also an improvement in the CSF findings during the course (cell count, total protein, quotient IgM, and albumin); the CSF-specific OCB turned negative on day 237. Not unexpectedly, an increase in neurofilaments in plasma and cerebrospinal fluid was observed after the end of tofersen therapy. MRI of the cervical and thoracic spine (day 196) showed similar findings as on day 126. A further MRI was not performed during the course.

A resumption of the injections with tofersen was not carried out. Adjusted regimens (reduced tofersen dose, possibly with accompanying prednisolone pulse therapy) were rejected by the patient.

The case shows that the occurrence of myelitis is a rare, but noticeable side effect of therapy with tofersen. Rising CSF cell counts and rising total protein should lead to closer observation of the patient, and attention should be paid to indications of transient weakness after the injections.

Antisense oligonucleotides (ASOs) represent an innovative and promising new form of therapy for many neurological disorders, particularly neurodegenerative diseases. However, there is some evidence that there is a group effect with immunological or inflammatory responses and consequent complications with the use of these classes of agents. For example, some cases of aseptic meningitis and (secondary) communicating hydrocephalus have been observed in the use of the experimental ASO tominersen in Huntington disease [11] as in the treatment of spinal muscular atrophy with the ASO nursinersen [1]. In a retrospective analysis, a fourfold increased risk of this complication has been calculated in SMA patients [12]. Pathophysiologically, an exaggerated intrathecal immune response needs to be discussed and further investigations are required to elucidate the underlying immunological mechanisms and predictive factors for future risk stratification in the treatment of affected patients.
